# Laboratory Evaluation and Field Testing of Dengue NS1 and IgM/IgG Rapid Diagnostic Tests in an Epidemic Context in Senegal

**DOI:** 10.3390/v15040904

**Published:** 2023-03-31

**Authors:** Oumar Ndiaye, Kevin Woolston, Aboubacry Gaye, Cheikh Loucoubar, Michael Cocozza, Cheikh Fall, Fatou Dia, Emily R. Adams, Marième Samb, Diogop Camara, Bacary Djilocalisse Sadio, Cheikh T. Diagne, Manfred Weidmann, Oumar Faye, Joseph R. A. Fitchett, Amadou Alpha Sall, Cheikh Tidiane Diagne

**Affiliations:** 1 Diatropix Unit, Institut Pasteur de Dakar, Dakar BP 220, Senegal; oumar.ndiaye@pasteur.sn (O.N.); fatou.dia@pasteur.sn (F.D.); marieme.samb@pasteur.sn (M.S.); 2 Virology Department, Institut Pasteur de Dakar, Dakar BP 220, Senegal; diogop.camara@pasteur.sn (D.C.); bacary.sadio@pasteur.sn (B.D.S.); oumar.faye@pasteur.sn (O.F.); amadou.sall@pasteur.sn (A.A.S.); 3 Global Access Diagnostics, Bedford Technology Park, Bedford MK44 2YA, Bedfordshire, UK; kevin.woolston@globalaccessdx.com (K.W.); michael.cocozza@mologic.co.uk (M.C.); emily.adams@globalaccessdx.com (E.R.A.); 4 Epidemiology Clinical Research and Data Science Department, Institut Pasteur de Dakar, Dakar BP 220, Senegal; aboubacry.gaye@pasteur.sn (A.G.); cheikh.loucoubar@pasteur.sn (C.L.); 5 Microbiology Department, Institut Pasteur de Dakar, Dakar BP 220, Senegal; cheikh.fall@pasteur.sn; 6 Mivegec Infectious Diseases and Vector: Ecology, Genetics, Evolution and Control, Université Montpellier, IRD, CNRS, 34394 Montpellier, France; c.diagne@icloud.com; 7 Institute of Microbiology and Virology, Medical School Brandenburg Theodor Fontane, D-01968 Senftenberg, Germany; manfred.weidmann@mhb-fontane.de; 8 Delivery Unit, Institut Pasteur de Dakar, Dakar BP 220, Senegal; joe.fitchett@pasteur.sn

**Keywords:** dengue, rapid tests, diagnostics, outbreak response, arboviruses

## Abstract

In Senegal, the burden of dengue is increasing and expanding. As case management and traditional diagnostic techniques can be difficult to implement, rapid diagnostic tests (RDTs) deployed at point of care are ideal for investigating active outbreaks. The aim of this study was to evaluate the diagnostic performance of the Dengue NS1 and Dengue IgM/IgG RDTs on the serum/plasma samples in a laboratory setting and in the field. During laboratory evaluation, performance of the NS1 RDT was assessed using NS1 ELISA as the gold standard. Sensitivity and specificity were 88% [75–95%] and 100% [97–100%], respectively. Performance of the IgM/IG RDT was assessed using the IgM Antibody Capture (MAC) ELISA, indirect IgG, and PRNT as gold standards. The IgM and IgG test lines respectively displayed sensitivities of 94% [83–99%] and 70% [59–79%] and specificities of 91% [84–95%] and 91% [79–98%]. In the field, the Dengue NS1 RDT sensitivity and specificity was 82% [60–95%] and 75% [53–90%], respectively. The IgM and IgG test lines displayed sensitivities of 86% [42–100%] and 78% [64–88%], specificities of 85% [76–92%] and 55% [36–73%], respectively. These results demonstrate that RDTs are ideal for use in a context of high prevalence or outbreak setting and can be implemented in the absence of a confirmatory test for acute and convalescent patients.

## 1. Introduction

Dengue fever is the most common arboviral fever in the world with an annual global incidence of 400 million cases, of which 96 million are symptomatic [1]. The dengue virus belongs to the genus *Flavivirus* within the *Flaviviridae* family. It is transmitted by *Aedes* mosquitoes, mainly *Aedes aegypti* or *Aedes albopictus* [2]. Dengue fever (DF) occurs abruptly after approximately 3 to 10 days of incubation with the onset of a high fever [3], often accompanied by headache, nausea, vomiting, joint and muscle pain, and a rash resembling measles [4]. Classic dengue, despite being highly disabling, is not considered a severe disease as patients recover generally between 3 and 7 days after the onset of symptoms [5]. However, it can progress to dengue haemorrhagic fever (DHF) and dengue with shock syndrome (DSS), which affect approximately 500,000 people annually [6], and without proper intensive care supportive treatment, the case fatality rate can be as high as 20% [7]. There are four dengue virus serotypes: DENV-1, DENV-2, DENV-3, and DENV-4 [8]. Each of the four serotypes is likely to produce an infection that can express the different clinical forms of the disease, but neither can induce cross-protection against the other serotypes in infected individuals [9]. A fifth sylvatic serotype has been described in Malaysia, but detailed data have not been published [10] nor has this been confirmed by other reports. Epidemiological studies show that the occurrence of severe forms such as DSS or DHF is more common with a secondary infection of a DENV serotype different from the one that caused the first infection. It has also been linked to the phenomenon of antibody dependent enhancement (ADE) [11,12,13].

In Senegal, the first strain of DENV-2 isolated from humans was reported in 1970 from Bandia (Thies region) [14]. Subsequently, multiple isolations of the virus from human sera, mosquitoes, and non-human primates have been reported [15]. The first documented outbreak of dengue fever in Senegal was reported in 1990 with virus isolated from human cases [16]. In 2009, a DENV3 outbreak erupted in the capital Dakar with a total of 196 confirmed DF and five DHF cases [17]. Since then, sporadic cases and regular outbreaks have been reported. In 2014, an epidemic of DF occurred in the Dakar and Mbour regions [18], and in 2017, a major outbreak of DF was recorded in the centre of the country at Louga [18]. Recently, in 2018, a major outbreak of DF was reported in three regions of the country [18] with the central region of Diourbel being the hardest hit during the annual pilgrimage of the Touba Magal [19]. Globally, a rapid increase in the number of dengue outbreaks associated with the co-circulation of several DENV serotypes have been reported in Senegal [20], thus increasing the incidence of severe cases due to ADE [21]. This might potentially pose a new serious public health threat in Senegal in the coming years. As there is no effective treatment against DF [22] and since the disappearance of the virus is unlikely due to its maintenance in host vectors and the existence of animal reservoirs, prevention is the only effective means of control [23]. As there is no effective vaccine available to the African market yet [24], prevention is based on the implementation of control programmes (epidemiological surveillance), which in turn relies heavily on time-consuming and expensive laboratory diagnostic tools to enable the detection of suspected cases for the early containment of the outbreaks [25]. Current laboratory diagnostics for DENV are based on direct methods aiming to detect the genomic RNA, the virus or non-structural protein 1 (NS1) and indirect methods detecting specific antibodies [26]. The detection of IgM/IgG antibodies for acute and convalescent patients is achieved through enzyme linked immunosorbent assay (ELISA) tests (IgM Antibody Capture ELISA for IgM and indirect IgG ELISA for IgG). However cross reactivity is a frequent issue in flavivirus ELISA tests, especially between DENV, YFV, ZIKV, and WNV [27]. False positives are also regularly reported with malaria positive samples [28]. Therefore, since Senegal and sub-Saharan Africa are endemic to flaviviruses and malaria, all ELISA results have to be confirmed by plaque reduction neutralisation tests (PRNTs) [29], which are highly specific. Viral isolation can be useful but is mainly used for research purposes [30], and the detection of viral genomic RNA is performed through RT-PCR for acute cases [31]. Secreted NS1 in plasma has also been found to be a remarkable biomarker for the detection of acute cases as it is found circulating from the first day of symptom onset to 9 days after, with higher levels observed in primary rather than in secondary infections [32]. These methods are currently not available in peripheral health care centres, take a long time to generate results and pose challenges regarding biosafety requirements. Therefore, they can only be undertaken by specialised central laboratories, thus creating delays prior to confirmation of an outbreak and delaying patient care.

Implementation of user-friendly rapid diagnostic tests (RDTs) deployed at point of care combined with real-time data collection is the key to effective disease surveillance. Commercial kits have been developed in the recent years for the detection of DENV infection in serum/plasma or whole blood, but a number of them still have low specificity for IgM and IgG antibody detection or require a high volume for the detection of dengue NS1.

In this paper, we describe the laboratory evaluation and field deployment of two novel immunochromatographic rapid diagnostic test prototypes co-developed by Global Access Diagnostics (GADx) and the Institut Pasteur de Dakar: a dual line test for the detection of IgM and IgG antibodies to dengue virus (DENV1-to DENV4), and a single line test for the detection of dengue NS1 for acute cases. The results were then compared with the current standard methods being used to diagnose dengue [29]. This evaluation was performed in two steps: a retrospective clinical evaluation took place in a BSL2 central lab in Dakar, Senegal on well-characterised bio-banked samples in June 2018, and a second evaluation during a longitudinal study was performed in the field in Touba city during the 2018 dengue outbreak in November–December 2018.

## 2. Materials and Methods

### 2.1. Ethical Statement

Bio-banked human samples or viral isolates used in this study are part of the Institut Pasteur de Dakar (IPD) collection of the World Health Organisation Collaborating Centre (WHOCC) for Arboviruses and Haemorrhagic Fever Viruses. Samples used in Touba were collected as part of the IPD outbreak management mission in Touba appointed by the Senegalese Ministry of Health according to Circular 0035 of 24 September 2018. All the samples were anonymised prior to use and analysis.

### 2.2. Laboratory Tests

#### 2.2.1. qRT-PCR for the Detection of Dengue Virus

Viral RNA was extracted under BSL-2 conditions from 140 µL of human serum the using Qiagen RNA Extraction Kit protocol (Qiagen, Hilden, Germany) as previously described [33]. According to the manufacturer’s instructions, in brief, 560 µL of viral lysis buffer AVL was added to the serum samples. After a 10 min incubation step at RT, the lysate was bound to a spin filter membrane and washed twice with Wash Buffers 1 and 2, then the RNA was eluted using 60 µL of elution buffer. The detection of the genomic RNA was carried using a Taqman reverse transcription specific degenerate forward primer (DEN FP), reverse primer (DEN RP), and probe (DEN P): DEN FP 5′AAggACTAgAggTTAKAggAgACCC3′, DEN RP 5′ggCCYTCTgTgCCTggAWTgATg3′, and the probe DEN P 5′ FAM-AACAgCATATTgACgCTgggARAgACC-TAMRA-3′, respectively. Forty amplification cycles were performed using an ABI 7500 fast thermocycler (Thermo Fisher Scientific, Waltham, MA, USA).

#### 2.2.2. Platelia Dengue Bio-Rad NS1 ELISA Test

All samples used for the Dengue NS1 RDT evaluation that had previously been tested by qRT-PCR were tested again by the enzyme-linked immunosorbent assay (ELISA) NS1 antigen test. The test was performed using the Platelia Dengue NS1 antigen capture test (Biorad Laboratories, Marnes-la-coquette, France) as the reference method according to the manufacturer’s instructions. The sample results were classified as follows using the optical density (OD) ratio: OD ratio < 0.5: negative, OD ratio 0.5–1.0: equivocal, OD ratio 1–5: low positive, OD ratio 5–6: medium positive, and OD ratio ≥ 6: high positive.

#### 2.2.3. IgM Antibody Capture (MAC)-ELISA

All samples used for the evaluation of the IgM test line were tested by MAC ELISA as the reference method. As described in [34], 96-well microtiter plates (Immulon II 96-well microtiter plates; Dynatech laboratories, Inc, El Paso, TX, USA) were coated with a monoclonal IgM capture antibody (Seracare, Milford, CT, USA) in carbonate bicarbonate buffer (pH 9.6) and incubated overnight at 4 °C. After an initial 1 h blocking step using 400 µL of phosphate-buffered saline plus 0.05% Tween-20 and 5% non-fat milk, plates were washed 4 times with wash buffer using phosphate-buffered saline plus 0.05% Tween-20. We then added the patient serum samples and controls (all diluted 1:100 in dilution buffer composed of phosphate-buffered saline plus 0.05% Tween-20 and 1% milk powder) in duplicate into plate wells and incubated at 37 °C for 1 h. We washed wells 4 times with wash buffer, added 100 µL of suckling mouse brain DENV antigens and controls diluted at 1:40 in dilution buffer into plate wells, and incubated the plate for 1 h. After 4 washes, we added 100 µL of the 6B6C-1 flavivirus group-reactive monoclonal antibody (MAb) conjugated to horseradish peroxidase (HRP) at 1:6000 in dilution buffer. After an incubation time of 1 h at 37 °C and four subsequent washing steps, we added 100 µL of ready to use tetramethylbenzidine (TMB) substrate (Sigma Aldrich, Saint Louis, MO, USA) to the IgM conjugate complex and stopped the colour reaction using 100 µL of sulphuric acid solution (H_2_SO_4_) at 1 N. Reactions were measured using a MultiSKANN reader (Thermo Fisher Scientific, Waltham, MA, USA) at an absorbance of 450 nm with 620 nm as the passive reference. Ratios between the optical density of the sample viral antigen OD and the negative control was used, any ratio (P) > 2 was considered positive after a negative background check. Readouts were as follows: OD ratio < 3 negative, OD ratio 3–7.5 low positive, OD ratio 7.5–15 medium positive, and OD ratio ≥ 15 high positive.

#### 2.2.4. Indirect IgG ELISA

All samples used for the evaluation of the IgG test line evaluation were previously screened by in-house indirect IgG ELISA as previously described for Crimean Congo haemorrhagic fever virus [35].

Briefly, standard ELISA plates (Immulon II 96-well microtiter plates; Dynatech laboratories, Inc., El Paso, TX, USA) were coated with 100 µL of in-house prepared mouse hyper immune ascitic fluids specific to either YFV, DENV, WNV, and ZIKV at 1/1000 dilution in phosphate buffer saline (PBS) solution at 0.135 M and coated overnight at 4 °C. After the initial four washing steps with 300 µL of wash buffer (PBS 1X-Tween-20-0.05%), 100 µL of in-house prepared specific corresponding mouse brain antigens to either YFV, DENV, WNV, and ZIKV were diluted at 1/40 in dilution buffer (PBS 1X-Tween-20-1% skimmed milk) and incubated at 37 °C for 1 h. After a 1 h incubation time, plates were washed four times with 300 µL of wash buffer and 100 µL of the samples; negative and positive controls were added at 1/100 dilution in dilution buffer. After 1 h incubation at 37 °C and four washing steps with 300 µL of wash buffer and 100 µL of goat anti-Human IgG horseradish peroxidase conjugated (Seracare, Milford, CT, USA), diluted at 1 µg/mL in dilution buffer, was added to the plates. After 1 h of incubation at 37 °C and subsequent washing steps with 300 µL of wash buffer, specific binding was revealed by the addition of 100 µL of ready to use 3,3′,5,5′-tetramethylbenzidine (TMB) (Catalog number T0440-100 ML, Sigma-Aldrich, Saint Louis, MI, USA) and subsequently stopped using 100 µL of 2 N sulphuric acid (H_2_SO_4_). Plates were later read on the spectrophotometer at a 450 nm wavelength and 620 nm as the passive reference. Sera were considered positive if the optical density was >0.20 above the negative sera average and the ratio (R) between the sample and the negative control was >2.

#### 2.2.5. Plaque Reduction Neutralisation Test

The ELISA IgM and IgG positive samples were analysed for flavivirus-neutralising antibodies using PRNT as described in [29].

(a) *PRNT testing for ZIKV, WNV, and YFV*

Briefly, heat inactivated sera samples with positive and negative controls were diluted 2-fold starting at 1:10 in L15 medium (Thermo Fisher Scientific, Waltham, MA, USA). A total of 100 µL of the diluted samples were then mixed separately with equal volumes of medium containing 1000 PFU/mL (plaque forming units) of the reference strains. Virus/serum mixtures were then incubated for 1 h at 37 °C and later used to infect the PS cell monolayers in 24-well plates. After 1 h of incubation at 37 °C, cells were covered with 400 µL of L15 medium containing 3% FBS (foetal bovine serum) and 0.4% carboxymethyl cellulose (CMC) and incubated for 4 days. Colouration of plaques was performed using blue-black dye solution (1% g/mL black amido, 1.36% g/mL sodium acetate, and 6% ml/mL acetic acid) staining. Briefly, after 4 days of incubation time, plates were washed with 400 µL of PBS 1X, and subsequently, 400 µL of a solution of the blue black dye solution was added to each well and incubated for at least 1 h. Plates were then washed with 400 µL of PBS 1X and left to dry under a Biosafety cabinet for at least 1 h. Neutralising antibody titres were determined using a 90% cut-off value and a sample was classified as positive if the titre was ≥1/20.

(b) *PRNT testing for DENV*

Neutralisation testing with DENV was performed using a focus reduction neutralisation assay (FRNT) as no lytic strains were available during the evaluation to perform a PRNT.

Briefly, heat inactivated sera samples with positive and negative controls were diluted 2-fold starting at 1:10 in DMEM (**Dulbecco’s modified Eagle medium**). A total of 100 µL of the diluted samples were then mixed separately with equal volumes of DMEM containing 800 FFU/mL (focus forming units) of the dengue 2 NGC reference strain. Virus/serum mixtures were then incubated for 1 h at 37 °C and later used to infect Vero cell monolayers in 24-well plates. After 1 h incubation at 37 °C, cells were covered with 400 µL of DMEM containing 3% FBS and 0.6% CMC and incubated for 6 days. Detection of dengue focus units was performed using aminoethylcarbazole (AEC) colouration. Briefly after 6 days of incubation, each well of the plates was fixed with 1 mL of a 1:1 solution of methanol and acetone. After an incubation at −20 °C for 30 min, the plates were washed with 400 µL of PBS 1X and blocked with 250 µL of PBS 1X-3%FBS solution. After 30 min of gentle shaking, 400 µL of dengue 2 virus specific mouse ascitic fluid diluted at 1/1000 in PBS 1X was added in each well. After 1 h of incubation, the plates were washed with 400 µL of PBS 1X, and subsequently, 250 µL of goat anti-mouse IgG HRP conjugated (Seracare, Milford, CT, USA) diluted in PBS 1X-FBS 3% at 1 µg/mL was added to the plates. After 1 h of incubation, plates were washed with 400 µL PBS 1X and 400 µL of prediluted aminoethylcarbazole solution (ENZO, Farmingdale, NY, USA) at 1:7 in a specific buffer provided by the manufacturer was added to the wells. After a 1 h incubation, the plates were washed and left to dry under a Biosafety cabinet for at least 1 h. Neutralising antibody titres were determined using a 90% cut-off value and a sample was classified as positive if the titre was ≥1/20.

##### Samples Description

- **Samples used for Dengue NS1 RDT lab evaluation**

For the evaluation of sensitivity and specificity, a set of 176 samples was used. For sensitivity, 48 samples from the Dengue RT-PCR and Bio-Rad NS1 positive patients representing all three serotypes circulating in Senegal were used. DENV4 positive serum was not available during the evaluation as no previous case had been reported in Senegal. For the specificity testing, 128 negative RT-PCR/Bio-Rad NS1 dengue samples were used; the distribution is given in Table 1.

Spiked samples were produced by spiking the virus supernatant produced in the C636 (*Ae. albopictus*) cell line culture into negative human serum samples at 1:1000. The spiked samples were later confirmed positive by RT-PCR. P. falciparum samples were obtained from tick drop positive samples confirmed by real-time PCR.

- **Samples used for Dengue IgM/IgG RDT lab evaluation**
  ✓ IgM RDT Evaluation Panel

For the evaluation of the sensitivity and specificity, a set of 187 human serum samples was used as described in Table 2. For sensitivity, 50 MAC ELISA positive samples confirmed by PRNT were used. For specificity testing, 137 negative MAC ELISA negative samples were used. Aside from closely related flaviviruses and malaria, rheumatoid factor positive samples were added as it has been proven that they do sometimes yield false positives in antibody assays [36].

✓ IgG RDT Evaluation Panel

For the evaluation of sensitivity and specificity, a set of 171 samples was used. For sensitivity, 39 healthy human donor serum samples and 50 convalescent samples from recently infected people were collected. The samples that tested positive by the Dengue IgG-ELISA were confirmed by the qualitative plaque reduction neutralisation test. For specificity testing, 82 samples that tested negative by the Dengue IgG-ELISA were used. For flaviviruses IgG positive samples (YFV and ZIKV), samples were confirmed by plaque reduction neutralisation tests. Table 3 describes the distribution of the samples and their characterisations:

All of these clinical samples were received and tested by the WHO Collaborating Centre for Arboviruses and Viral Haemorrhagic Fevers within the Virology Department of the Institut Pasteur de Dakar.

- **Samples used for field evaluation**

A total of 88 serum samples were collected from Touba Central Hospital that corresponded to the selected case definition: fever > 38 °C with two or more of the following symptoms (headache, myalgia, arthralgia, rash, retro-orbital pain, haemorrhagic manifestations, or neurological signs). Forty-six acute samples (with date of collection < 10 days’ post onset disease symptoms (dpo)) were screened by RT-PCR prior to Dengue NS1 RDT testing. All 88 samples were tested with the Dengue IgM/IgG RDT and then later confirmed by MAC ELISA and plaque reduction neutralisation tests back to the WHO Collaborating Centre on Arboviruses and Viral Haemorrhagic Fevers in IPD.

#### 2.2.6. Dengue Rapid Diagnostic Tests Used for Evaluation

- **Specimen collection, transportation, and processing**

Venous whole blood is collected by venepuncture in either clot activator tubes for serum or in an anticoagulant (EDTA, heparin, or citrate) for plasma collection. The tubes were stored at 2–4 °C prior to shipment under cold chain to the laboratory. After reception, samples were centrifuged at 4000 rpm for 5 min and the serum/plasma collected.

- **Dengue NS1 Rapid Diagnostic Test**

**Figure 1:**
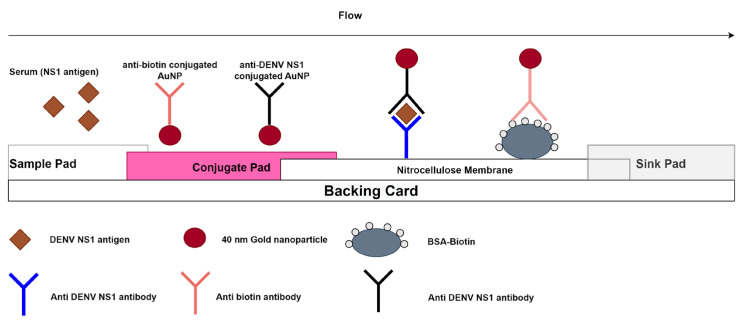
Schematic illustration of the Dengue NS1 rapid antigen test.

The Dengue NS1 RDT is a lateral flow immunoassay for the detection of DENV NS1 in human sera. The test strip has one test line and one control line as illustrated in Figure 1.

In brief, 40 µL of sample is deposited on the sample pad, then 10 s later, 40 µL of running buffer is added to the well. If NS1 antigen is present in the sample, it binds to the anti-DENV-NS1 antibodies specific to all serotypes coated on the 40 nm gold nanoparticles when passing through the conjugate pad. The antigen–antibody–gold nanoparticle complex then flows through the nitrocellulose (NC) membrane after the addition of 40 µL chase buffer onto the sample pad. The NS1 antigen will bind to the anti-DENV-NS1 antibody present on the test line and result in a red line. If the specimen does not contain the DENV-NS1 antigen, labelled complexes do not bind at the test zone and a line is not observed. The remaining colloidal gold flows up to the control line, where anti-biotin gold nanoparticles will bind to the BSA-biotin. A red line must appear at the control line zone for all valid tests whether the sample is positive or negative (Figure 1).

After a 10-min migration time; a colour intensity card was used to read the intensity of the test lines, which ranged from score 0 to 10 (Figure S1 in the Supplementary Materials). Results were visually read by one operator in order to avoid variability in the interpretation. A value of 0 was considered negative and any visible line ≥1 was considered positive.

The results were interpreted through the following algorithm:

✓ A red control line and NS1 test line indicate a positive result for DENV-NS1 antigen;
✓ A red control line alone indicates a negative result for DENV-NS1 antigen;
✓ Any result without a red control line is invalid.

Figure S2 in the Supplementary Materials presents a more detailed snapshot of the test results.

- **Dengue IgM/IgG Rapid diagnostic test**

**Figure 2:**
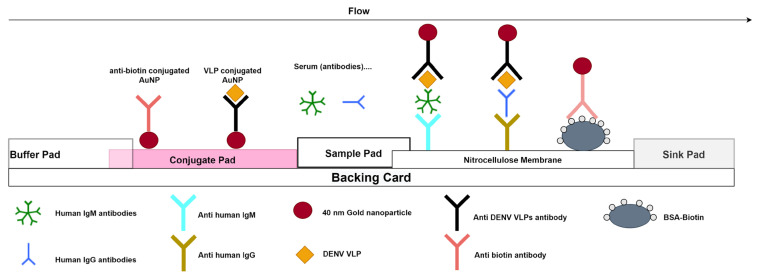
Schematic illustration of the Dengue IgM/IgG RDT test.

The Dengue IgM/IgG RDT is a lateral flow immunoassay for the detection of anti-DENV IgM and IgG antibodies in human sera. The test strip has two test lines (IgM and IgG) and one control line as illustrated in Figure 2.

Briefly, 10 µL of the sample is deposited on the sample pad (well A) and then migrates through the NC strip, when IgM or IgG are present in the sample; they bind to the anti-human IgM or anti-human IgG antibodies immobilised on the nitrocellulose membrane. After 10 s, 80 µL of running buffer is added to well B corresponding to the buffer pad, which then flows through the conjugate pad releasing the virus like particles (VLPs) of the four different serotypes coated on the 40 nm gold nanoparticles; the conjugated antibodies then flow from the conjugate pad and pass through the NC membrane. Specific IgM or IgG antibodies previously bound on the NC strip will bind to the gold coated VLPs, creating a red line. If the specimen does not contain DENV specific antibodies, labelled conjugates will not bind at the test zone and M or G lines are not observed. The remaining colloidal gold flows up to the control line, where anti-biotin gold nanoparticles will bind to the BSA-biotin. A red line must appear at the control line zone for all valid tests, regardless of whether the sample is positive or negative (Figure 2). After a 10-min migration time, the results were interpreted through the following algorithm:

✓ A red control line and IgM test line indicate a positive result for anti-dengue IgM;
✓ A red control line and IgG test line indicate a positive result for anti-dengue IgG;
✓ A red control line and both test lines (IgM and IgG) indicated a positive result for both anti-dengue IgM and anti-dengue IgG;
✓ A red control line alone indicates a negative result;
✓ Any result without a red control line indicates an invalid test.

Results were visually read by one operator in order to avoid variability in the interpretation.

Figure S2 in the Supplementary Materials provides a more detailed snapshot of the test results.

## 3. Results

### 3.1. Central Laboratory Evaluation Results

#### 3.1.1. Dengue NS1 Lateral Flow Test Results

**Figure 3:**
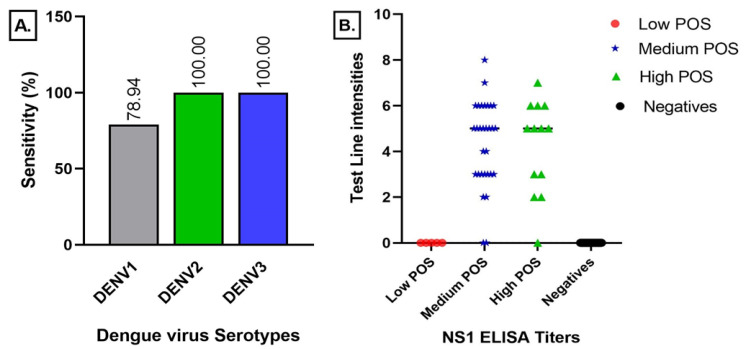
Correlation between the serotype versus visual scores (**A**) and ELISA NS1 ratios (**B**).

Of the 48 Platelia Dengue NS1 (Biorad Laboratories, Marnes-la-coquette, France) positive samples, 42 were scored positive by the Dengue NS1 RDT, yielding a sensitivity of 88% [75–95%]. All 128 negative samples (Table 1) were scored as negative, yielding a specificity of 100% [97–100%] across all agents tested. The serotype sensitivity of the Dengue NS1 RDT for DENV-1 was found to be the lowest at 79% [63–90%] while it was 100% [59–100%] for DENV-2 and DENV-3 [29–100%] (see Figure 3), however, they had lower sample populations (Table 1). Specificity was determined at 100% [97–100%] for all of the conditions tested. Table S1 in Supplementary Materials provides exhaustive detailed results.

#### 3.1.2. Dengue IgM Lateral Flow Test Results

**Figure 4:**
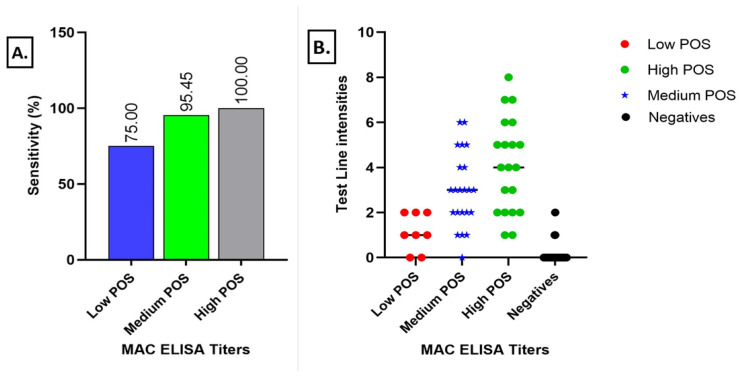
Sensitivity of the IgG/IgM RDT versus ELISA IgM ratio (**A**) and MAC ELISA titres (**B**).

Of the 50 MAC-ELISA & PRNT positive samples, 47 were confirmed as positive by the Dengue IgM/IgG RDT, yielding a sensitivity of 94% [83–99%]. It also scored 124/137 dengue IgM/IgG as negative samples, yielding a specificity of 91% [84–95%], (Table S2 in the Supplementary Materials). Sensitivity with grouped dengue samples was 75% [35–97%], 95% [77–100%], and 100% [83–100%] for low, medium, and high positive samples, respectively, which was also reflected in the distribution of the band intensity of the RDT result lines (Figure 4).

#### 3.1.3. Dengue IgG Lateral Flow Test Results

**Figure 5:**
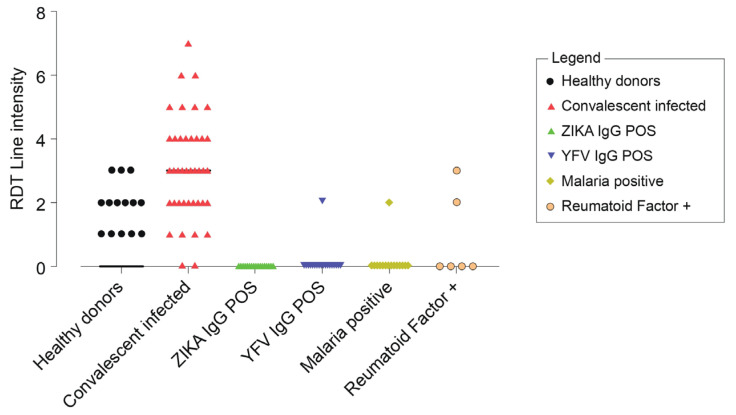
Sensitivity and specificity of IgG test line.

A sensitivity of 70% [59–79%] was found with the sensitivity panel samples. A specificity of 91% [79–98%] was found with cross reacting flaviviruses (ZIKV, YFV) and interfering conditions (malaria, rheumatoid factor). See Table S3 in the Supplementary Materials for exhaustive detailed results. Figure 5 shows the distribution of the test line intensities depending on the sample characterisation.

### 3.2. Field Evaluation Results

Among the 46 suspected acute samples (date of onset <10 days), 22 samples were found to be positive by Dengue RT-PCR, among which 18 were positive with the Dengue NS1 RDT, giving a sensitivity of 82% [60–95%], and six samples with low intensity test lines were found positive with the Dengue NS1 RDT but negative by RT-PCR, yielding a specificity of 75% [53–90%].

Among the 88 samples tested for antibodies, seven were IgM positive by the MAC-ELISA and PRNT test and 6/7 were scored positive by the Dengue IgM/IgG RDT, resulting in a sensitivity of 86% [42–99%] and a specificity of 85% [76–92%]. Indirect ELISA and PRNT confirmed 54/88 positive for IgG, of which 42 were found positive by the Dengue IgM/IgG RDT, resulting in a sensitivity of 78% [64–88%] and the specificity was found to be 55% [36–73%] (Cf. Table S4 in the Supplementary Materials).

## 4. Discussion

Early detection of dengue cases is critical for patient management in tropical areas where the clinical symptoms of dengue fever can easily be confused with other flu-like symptoms [37]. The accuracy of diagnostic methods depends on the collection time of the sample from the onset of disease [26]. Cold chain dependency is also important for most molecular techniques (e.g., to preserve viral RNA). Therefore, in low- and middle-income countries (LMICs), where access to electricity or rapid transportation of patient samples can be delayed, rapid diagnostic tools are ideal for effective on site diagnostics and patient management at point-of-care and can be easily implemented in low resource settings [38].

In this study, we evaluated the performance of a prototype Dengue NS1 RDT for the detection of acute cases and a prototype Dengue IgM/IgG RDT for the detection of both acute and convalescent cases in two different settings: first in a central laboratory setting, followed by an evaluation in the field at the patient level during an active outbreak of dengue virus in Touba city, a city located in central Senegal. A high sensitivity of 88% [75–95%] was observed with the Dengue NS1 RDT during lab evaluation compared to the ELISA NS1 gold standard. Analysis of the correlation between the band intensity and ELISA ratios showed that ratios under 3.49, which correspond to low positives, were missed by the Dengue NS1 RDT, however, the majority of specimens with a ratio of more than 4 were picked up on the test line. Serotype sensitivity was shown for DENV1-3 but not confirmed for DENV4 due to the lack of DENV-4 positive samples from Senegal. Despite the number of true positives across the three serotypes being low, we are confident that this initial data indicate that all three serotypes could be reliably detected. Further investigations into the relationship between sensitivity and dpo as well as the type of infection (primary or secondary dengue) will help to grasp the full extent of the sensitivity of the test; as with DF, early identification is crucial in order to help in patient triage, and the timely management of the infection can prevent it from evolving into DHF or DSS [39], which can both be fatal. Importantly, no false positive cross detection was observed, yielding a high specificity of 100% [97–100%], especially for the closely related flaviviruses ZIKV and YFV.

A sensitivity of 82% [60–95%] and a specificity of 75% [53–90%] for the Dengue NS1 RDT was achieved with field samples, resulting in NPV and PPV values of 82% [64–92%] and 75% [59–86%], respectively. Although these values indicate a lower specificity compared to the RT-PCR, it must be noted that since the NS1 ELISA chain cannot be deployed in the field due to its relative complexity, RT-PCR was used as a reference and might have an impact in the true number of dengue NS1 positive cases. This might be due to the fact that the dengue NS1 protein lingers longer in the organism compared to viral RNA, which is more labile; as described in a previous study, dengue NS1 can be found up to 9 days post infection [40], thus the NS1 RDT might be picking more true positives than the RT-PCR, especially among samples with very low viremia that become undetectable by RT-PCR but still have a detectable amount of NS1 protein. Taking the latter fact into account, this RDT proves that it might be a valuable tool in an acute arboviral fever outbreak with multiple co-circulating flaviviruses as its availability and time to result potentially have a greater impact than their reduced performance characteristics [41] compared to RT-PCR or NS1 ELISA.

Compared to the RDTs already on the market as described in [42], where the respective sensitivities obtained from three different manufacturers were 63%, 48.62%, and 79.82%, one can see that the prototype described here displayed a higher performance both during laboratory evaluation and in the field.

The IgM/IgG RDT showed good performances during lab evaluation for the IgM test line with a sensitivity of 94% [83–99%]. Sensitivity per MAC ELISA titre showed the best sensitivity for high positive samples at 100% [83–100%], followed by medium positives with 95% [77–100%] and low positive samples with 75% [35–97%]. Analysis of the correlation between the MAC ELISA titres and the visual score showed a strong link (p value at 0.0008).

In a next step, the correlation between neutralising antibodies titres and the IgM test line intensities needs to be investigated. A high specificity of 91% [84–95%] has been found for the IgM test line, with little cross reactivity noted for YFV-IgM but moderate cross reactivity for ZIKV-IgM. Compared to traditional methods, these results are very encouraging, marking a clear important improvement in the serological diagnostics of dengue virus disease, which is often impaired by flavivirus cross reactivity. Such high specificity means that the test could be used without the need to use a PRNT for confirmation of DENV-IgM.

During the field evaluation, the IgM sensitivity and specificity were quite high, at 86% [42–99%] and 85% [76–92%], respectively. A high NPV was also found at 99% [92–100%], which indicates that the test discriminated well between dengue infections and other similar febrile aetiologies. PPV was low at 33% [13–59%], which might be due to the low number of samples tested during the study (six samples) and the low prevalence of the antibodies, as most of the patients during the study period tended to turn up early to the health centre.

Compared to RDTs already on the market as described in [43], where the sensitivities obtained from six different manufacturers ranged between 70% and 79%, the specificities were between 46.3% and 89.4%. Given these results, the prototype we describe here displays a higher performance for the detection of IgM antibodies during both the laboratory evaluation and in the field.

For the IgG test line, sensitivity was found to be at 70% [59–79%], which was relatively low compared to the IgM test line, especially among the healthy patients. Low IgG sensitivity detected in healthy donors (Figure 5) may be due to low avidity IgG antibodies present at low levels in sera after recent asymptomatic DF infection. Three quarters of all DF cases are assumed to be asymptomatic [44] and low avidity IgG after recent infection have been described for WNV and DF [45]. IgG antibodies of low avidity are efficiently detectable by indirect ELISA but less so by lateral flow assays. Different factors could explain this phenomenon. In ELISA, the different steps and long incubation time allow for greater molecular binding of the different molecules, therefore increasing the sensitivity of the test. In lateral flow, all of the molecular interactions have to take place within 10 min and during the migration, so low avidity antibodies might bind weakly to the antigens, thus generating only a faint test line. Field results obtained during the Touba outbreak seem to confirm this hypothesis as the IgG sensitivity was 78% [64–88%] among the convalescent serum samples that were dual positive samples by ELISA (IgM and IgG). For the IgG test line, a high specificity was reported during the laboratory evaluation with 91% [79–88%], and cross reactivity was not observed with the Zika IgG virus positive samples whereas 1/10 YFV IgG samples that were negative in the DENV-ELISA test scored positive on the IgG test line. Two samples appeared positive with the rheumatoid factor panel. Indeed, the highest cross reactivity of DENV IgG-antibodies is known to be for YFV [46] then for ZIKV and DENV [27].

Given the performances obtained with the NS1 RDT, which detects acute cases, and IgM test line, which detects acute and convalescent cases, the combination of these tests proved suitable for the testing of active cases or for seroconversion surveys, but might not be for seroprevalence studies among healthy persons.

Current serological diagnostics use a combination of MAC-ELISA and PRNT for the confirmation of suspected cases, and the results can be obtained in about 5 days, therefore implementing this RDT, which is cheap, easy to use, requires less sample volume, and hands on time, might significantly reduce the confirmation time to just a matter of minutes and greatly increase the timeliness of any response during an outbreak.

Slight differences might be observed between operators for antibody testing based on visual interpretation of the gold intensity card, so using an automatic reader could enhance the reliability of the interpretation of the test results, especially for low positive samples. In the case of the unavailability of a specific reader, visual inspection by three different operators should be considered. Potential limitations of this study are (1) the absence of replicability/reproducibility evaluation in another lab/field setting and (2) only serum/plasma samples were evaluated mainly due to bio-banking issues. Therefore, venous blood and centrifugation are still required prior to sample testing. Matrix equivalency studies are planned in the next stages of development to assess the use of capillary or venous whole blood.

## 5. Conclusions

Given the performances obtained during both the laboratory evaluation as well as the field deployment, these RDTs offer a clear advantage over more traditional methods that are lengthy and too costly. As the DF burden is increasing in Africa, deploying these tests for the active surveillance of suspect DF cases could contribute to strengthening arbovirus surveillance systems and outbreak control.

## Figures and Tables

**Table 1:** Dengue NS1 sensitivity and specificity panel.

Dengue NS1 Sensitivity Panel		Dengue NS1 Specificity Panel	
*Serotype*	n=	*Related flaviviruses*	n=
DENV1 natural infection	38	YFV spiked samples	25
DENV2 natural infection	7	ZIKV spiked samples	20
DENV3 natural infection	3	WNV spiked samples	19
TOTAL	48	*Other Febrile illnesses*	
		CHIKV spiked samples	20
		P. falciparum positive samples	34
		*Healthy donor samples*	
		NEG all *	10
		TOTAL	128 *

* NEG all samples were obtained from healthy persons tested negative by RT-PCR and IgG/IgM ELISA for Rift Valley fever (RVFV), Crimean Congo haemorrhagic fever (CCHFV), Yellow fever (YFV), DENV, West Nile (WNV), Chikungunya (CHIKV), and ZIKV.

**Table 2:** Dengue IgM sensitivity and specificity panel.

Dengue IgM Sensitivity Panel		Dengue IgM Specificity Panel	
DENV MAC ELISA Titre	n=	*Related flaviviruses*	n=
High POS	20	IgM YFV	41
Medium POS	22	IgM ZIKV	20
Low POS	8	*Other Febrile illnesses*	
TOTAL	50	Malaria positive	20
		Interfering Conditions	
		Rheumatoid Factor+	6
		*Healthy donor samples*	
		NEG all *	50
		TOTAL	137

* NEG all samples were obtained from healthy persons tested negative by RT-PCR and IgG/IgM ELISA for Rift Valley fever (RVFV), Crimean Congo haemorrhagic fever (CCHFV), Yellow fever (YFV), DENV, West Nile (WNV), Chikungunya (CHIKV), and ZIKV.

**Table 3:** Dengue IgG sensitivity and specificity panel.

**Dengue IgG Sensitivity Panel**		**Dengue IgG Specificity Panel**	
Serum description	n=	Serum description	n=
Healthy donor samples IgG POS	39	*Other Febrile illnesses*	
Convalescent IgG POS	50	Malaria PCR positive	6
		*Interfering Conditions*	
TOTAL	89	Rheumatoid Factor+	6
		*Healthy donor samples*	
		NEG all *	50
		*Related flaviviruses*	
		IgG YFV	10
		IgG ZIKV	10
		TOTAL	82

* NEG all samples were obtained from healthy persons tested negative by RT-PCR and IgG/IgM ELISA for Rift Valley fever (RVFV), Crimean Congo haemorrhagic fever (CCHFV), Yellow fever (YFV), DENV, West Nile (WNV), Chikungunya (CHIKV), and ZIKV.

## Data Availability

The data presented in this study are available within the article and the supplementary data provided.

## Supplementary Materials

The following supporting information can be downloaded at: https://www.mdpi.com/article/10.3390/v15040904/s1, Figure S1: Gold colour intensity card used for the evaluation; Table S1: Dengue NS1 RDT results; Table S2: Dengue IgM/IgG RDT IgM test line results; Table S3: Dengue IgM/IgG RDT IgG test line results; Table S4: Field evaluation results of the Dengue RDTs; Figure S2: Illustration of different serology testing results using the Dengue IgM/IgG RDT; Figure S3: Illustration of different test results using the Dengue NS1 RDT.
