# Supplementary material for: Laboratory Evaluation and Field Testing of Dengue NS1 and IgM/IgG Rapid Diagnostic Tests in an Epidemic Context in Senegal
Source: Viruses. 2023 Mar 31;15(4):904. doi: 10.3390/v15040904 (PMC10143717; doi:10.3390/v15040904)
Supplement: Supplementary file 1 [file viruses-15-00904-s001.zip › viruses-2104742-supplementary.pdf]

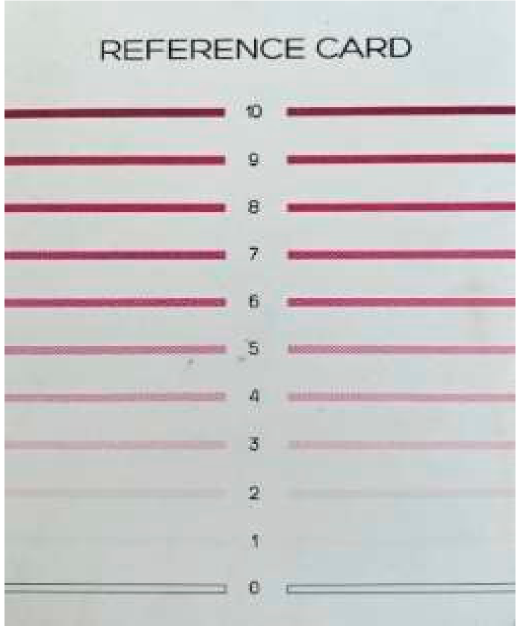

Figure S1. Gold colour intensity card used for the evaluation

Table S1. Dengue NS1 RDT Results

Panel Tested	Condition tested	reference test	analysed test	analysis values	Sensitivity (%)	Specificity (%)	n	analysed method	reference method pos	reference method Neg
Sensitivity Panel	All Dengue NS1 Panel	Biorad-NS1 ELISA	Dengue NS1 RDT	estimate:	88	100	176	pos	42	0
				95%CI:	[75-95]	[97-100]				
				p-value	1.01e-07	5.88e-39		neg	6	128
	Dengue 1 Serotype	Biorad-NS1 ELISA	Dengue NS1 RDT	estimate:	79		166	pos	30	0
				95%CI:	[63-90]					
				p-value	4.72e-04			neg	8	128
	Dengue 2 Serotype	Biorad-NS1 ELISA	Dengue NS1 RDT	estimate:	100		135	pos	7	0
				95%CI:	[59-100]					
				p-value	1.56e-02			neg	0	128
	Dengue 3 Serotype	Biorad-NS1 ELISA	Dengue NS1 RDT	estimate:	100		131	pos	3	0
				95%CI:	[29-100]					
				p-value	2.5e-01			neg	0	128
Specificity Panel	YFV spiked samples	Biorad-NS1 ELISA	Dengue NS1 RDT	estimate:		100	73	pos	42	0
				95%CI:		[86-100]				
				p-value		5.96e-08		neg	6	25
	ZIKV spiked samples	Biorad-NS1 ELISA	Dengue NS1 RDT	estimate:		100	68	pos	42	0
				95%CI:		[83-100]				
				p-value		1.91e-06		neg	6	20
	WNV spiked samples	Biorad-NS1 ELISA	Dengue NS1 RDT	estimate:		100	67	pos	42	0
				95%CI:		[82-100]				
				p-value		3.81e-06		neg	6	19
	CHIKV spiked samples	Biorad-NS1 ELISA	Dengue NS1 RDT	estimate:		100	68	pos	42	0
				95%CI:		[83-100]				
				p-value		1.91e-06		neg	6	20
	P. falciparum positive samples	Biorad-NS1 ELISA	Dengue NS1 RDT	estimate:		100	82	pos	42	0
				95%CI:		[90-100]				
				p-value		1.16e-10		neg	6	34
	NEG all *	RT-PCR	Dengue NS1 RDT	estimate:		100	58	pos	42	0
				95%CI:		[69-100]				
				p-value		1.95e-03		neg	6	10

Table S2. Dengue IgM/IgG RDT IgM test line results

Panel Tested	sample type	reference test	analysed test	analysis values	Sensitivity (%)	Specificity (%)	n	analysed method	reference method	
									pos	neg
Sensitivity	All Dengue	MAC	Dengue IgM	estimate:	94	91	187	pos	47	13
Panel	IgM	ELISA	test line	95%CI:	[83-99]	[84-95]				
	Panel	&PRNT		p-value	3.71e-11	6.84e-24		neg	3	124
	Dengue IgM	MAC	Dengue IgM	estimate:	100		157	pos	20	13
	High POS	ELISA	test line	95%CI:	[83-100]					
		&PRNT		p-value	1.91e-06			neg	0	124
	Dengue IgM	MAC	Dengue IgM	estimate:	95		159	pos	21	13
	Medium POS	ELISA	test line	95%CI:	[77-100]					
		&PRNT		p-value	1.1e-05			neg	1	124
	Dengue IgM	MAC	Dengue IgM	estimate:	75		145	pos	6	13
	Low POS	ELISA	test line	95%CI:	[35-97]					
		&PRNT		p-value	2.89e-01			neg	2	124
Specificity	IgM Yellow	MAC	Dengue IgM	estimate:		90	91	pos	47	4
Panel	Fever	ELISA	test line	95%CI:		[77-97]				
		&PRNT		p-value		1.03e-07		neg	3	37
	IgM ZIKA	MAC	Dengue IgM	estimate:		65	70	pos	47	7
		ELISA	test line	95%CI:		[41-85]				
		&PRNT		p-value		2.63e-01		neg	3	13
	Malaria posi-	MAC	Dengue IgM	estimate:		100	70	pos	47	0
	tive	ELISA	test line	95%CI:		[83-100]				
		&PRNT		p-value		1.91e-06		neg	3	20
	Rheumatoid	MAC	Dengue IgM	estimate:		100	56	pos	47	0
	Factor+	ELISA	test line	95%CI:		[54-100]				
		&PRNT		p-value		3.13e-02		neg	3	6
	NEG to all *	MAC	Dengue IgM	estimate:		92	100	pos	47	4
		ELISA	test line	95%CI:		[81-98]				
		&PRNT		p-value		4.46e-10		neg	3	46

Table S3. Laboratory evaluation of the Dengue IgM/IgG RDT IgG test line results.

Panel Tested	Condition tested	reference test	analysed test	analysis values	Sensitivity (%)	Specificity (%)	n	analysed method	reference method pos	reference method neg
Sensitivity Panel	All Dengue IgG Panel	IgG ELISA &PRNT	Dengue IgG Test Line	estimate:	70	91	121	pos	62	3
				95%CI:	[59-79]	[79-88]				
				p-value	<0.0001	<0.0001		neg	27	29
Specificity Panel	IgG Yellow Fever	IgG ELISA &PRNT	Dengue IgG Test Line	estimate:		90	99	pos	62	1
				95%CI:		[55-99]				
				p-value		0.0004		neg	27	9
	IgG ZIKA	IgG ELISA &PRNT	Dengue IgG Test Line	estimate:		100	99	pos	62	0
				95%CI:		[69-100]				
				p-value		<0.0001		neg	27	10
	Malaria positive	IgG ELISA &PRNT	Dengue IgG Test Line	estimate:		83	95	pos	62	1
				95%CI:		[35-99]				
				p-value		0.0156		neg	27	5
	Rheumatoid Factor+	IgG ELISA &PRNT	Dengue IgG Test Line	estimate:		67	95	pos	62	2
				95%CI:		[22-95]				
				p-value		0.0863		neg	27	4
	NEG all**	IgG ELISA &PRNT	Dengue IgG Test Line	estimate:		1	100	pos	47	0
				95%CI:		[93-100]				
				p-value		<0.0001		neg	3	50

Table S4. Field Evaluation results of the Dengue RDTs

reference test	analysed test	analysis values	Sensitivity (%)	Specificity (%)	PPV (%)	NPV (%)	n	analysed method	reference method pos	reference method neg
MAC-ELISA &PRNT	Dengue IgM Test Line	estimate:	86	85	33	99	85	pos	42	14
		95%CI:	[42-100]	[76-92]	[13-59]	[92-100]				
		p-value	1.25e-01	7.03e-11	2.38e-01	1.2e-19		neg	12	17
IgG ELISA &PRNT	Dengue IgG Test Line	estimate:	78	55	75	59	88	pos	6	12
		95%CI:	[64-88]	[36-73]	[62-86]	[39-76]				
		p-value	5.21e-05	7.2e-01	2.34e-04	4.58e-01		neg	1	69
Dengue RT-PCR	Dengue NS1 RDT	estimate:	82	75	75	82	46	pos	18	6
		95%CI:	[60-95]	[53-90]	[53-9]	[6-95]				
		p-value	4.34e-03	2.27e-02	2.27e-02	4.34e-03		neg	4	18

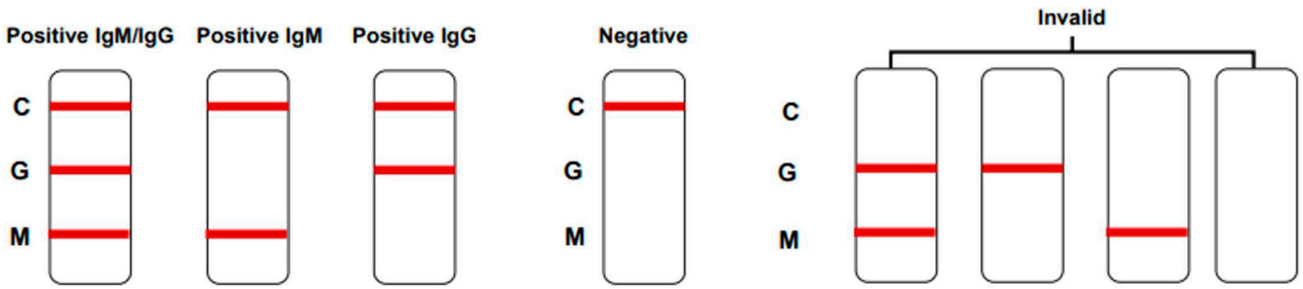

Figure S1. Illustration of different serology testing results using the Dengue IgM/IgG RDT.

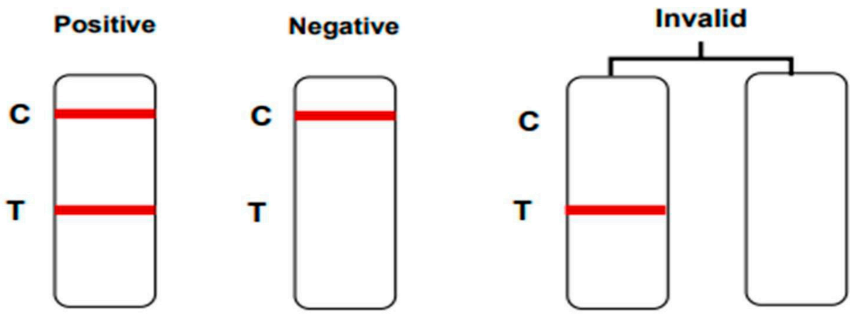

Figure S3. Illustration of different test results using the Dengue NS1 RDT
